# Secondary peritoneal hydatidosis, the challenges of echinococcal disease in South Sudan: a case report

**DOI:** 10.11604/pamj.2015.20.15.5809

**Published:** 2015-01-06

**Authors:** Richard William Thomas, Rwth Ellis-Owen, Daniel Winson

**Affiliations:** 1 General Medicine, Prince Philip Hospital, Llanelli, United Kingdom; 2 Consultant Radiologist, University Hospital of Wales, Cardiff, United Kingdom; 3 Core Surgical Trainee, University Hospital of Wales, Cardiff, United Kingdom

**Keywords:** South Sudan, hydatid, cystic echinococcus

## Abstract

A 28 year old male presented to the Juba Teaching Hospital with progressive shortness of breath. 18 months prior to admission, he presented to a rural hospital with severe abdominal pain. An emergency laparotomy was performed, and a large hepatic cyst was removed. Examination at the Juba Teaching hospital revealed a grossly distended abdomen with multiple palpable masses per abdomen. An Abdominal Ultrasound revealed multiple loculated cysts throughout the abdomen. A diagnosis of Secondary Peritoneal Hydatidosis resulting from incorrectly performed surgery was made. The patient was conservatively treated and at 14 weeks, the cysts showed a moderate reduction in size. Cystic Echinococcus(CE) is common in South Sudan and has a considerable disease burden throughout the developing world. Greater governmental and international support is required to develop effective control measures for these diseases.

## Introduction

Hydatid disease is a cyst forming condition resulting from infection by parasitic tapeworms of the Echinococcus genus. Most commonly the disease results from Echinococcus granulosa infection, termed cystic echinococcosis (CE), with the formation of unilocular cysts. A less common variant of the disease is caused by E. multilocularis, a more aggressive condition characterised by invasive multilocular cyst formation. In both forms, predominant primary hosts of the tapeworm are dogs and foxes; transmission to humans is via the faeco-oral route. The ova penetrate through the wall of the small intestine with subsequent haematogenous spread to the liver and other organs [1, 2]. Clinical manifestations depend on the site and size of the cyst. Approximately 70% and 20% of cysts resulting from E.granulosa infection are found in the liver and lungs respectively. Patients typically present with symptoms of mass effect. Rupture or infection of the cysts can lead to potentially fatal anaphylactic reactions, and as such these cysts require careful surgical and medical management. Prevalence of CE in South Sudan is known to be high. This is mainly attributed to the nomadic tribal population [3]. However a lack of basic medical facilities and diagnostic tools may further increase the burden of CE throughout East Africa.

## Patient and observation

**Figure 1:**
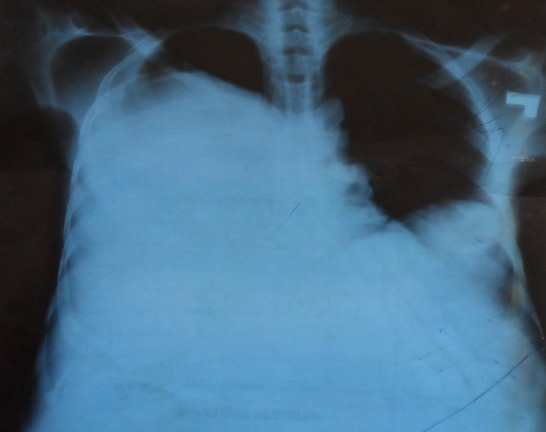
Chest X-Ray demonstrating an elevated right hemi-diaphragm with an accompanying small pleural effusion

**Figure 2:**
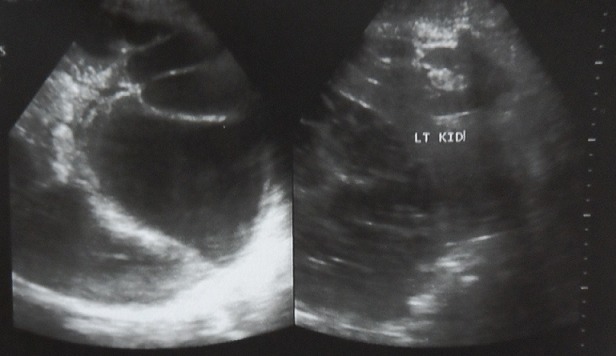
Ultrasound images of the liver shows a large dominant ‘mother cyst’ containing multiple septa between peripherally placed daughter cysts. This cyst-within-cyst appearance is also referred to as the ‘Spoke wheel sign’ or the ‘Honey comb sign’ and is typical of Hydatid Disease

Written consent was obtained from the patient prior to writing this case report. A 28 year old farmer from rural South Sudan presented to the emergency department of Juba teaching Hospital, complaining of shortness of breath and fatigue of 2 weeks duration. Eighteen months previously he had presented to a rural clinic in Yei complaining of abdominal pain and vomiting. Due to lack of access to Ultrasound and CT, an exploratory laparotomy was performed, finding a large hepatic cyst. The cyst was removed and the patient initially gained symptomatic relief. However, over the following six months, he complained of increasing abdominal distension, pain, weight loss and poor appetite. On examination, there were reduced breath sounds throughout the right lung with coarse left basal inspiratory crackles. The abdomen was grossly distended with multiple palpable masses and a large incisional hernia measuring 4cm x 6cm was present. The abdomen was non tender, with audible bowel sounds. A chest X-ray on admission revealed a massively elevated right hemi-diaphragm (Figure 1). Abdominal Ultrasound revealed multiple cysts of varying sizes throughout the abdominal and pelvic cavities, the largest cyst measuring 20cm * 19cm (Figure 2). Stool microscopy was positive for Echinococcal cysts. Bloods revealed a microcytic anemia, with a haemoglobin of 68. A diagnosis of secondary peritoneal hydatidosis and subsequent pneumonia was made. In the acute setting, the patient was transfused with red cells and given intravenous Co-Amoxiclav, his shortness of breath responding well to this initial treatment. He has now received 14 weeks of Albendazole and Metronidazole treatment, with the cysts showing a moderate reduction in size. With the current political situation throughout South Sudan the patient was lost to follow-up.

## Discussion

The prevalence of CE in humans and animals is high throughout East Africa, with a particular disease burden found in the nomadic pastoral tribes [4, 5]. In addition to the morbidity and mortality associated with the disease, CE incurs a considerable socio-economic cost worldwide. Budke et al (2006) estimated a loss of 1 million Disability-Adjusted life years worldwide, incurring an estimated total cost of $2 Billion [6]. The study went onto highlight the considerable economic impact of livestock losses resulting from CE. Despite its impact, CE remains on the WHO list of neglected tropical diseases [7]. This list comprises a collection of tropical diseases affecting mainly the poorest 1 Billion people. Their impact is enhanced by a lack of investment in research and disease control policies [8], further reinforcing the cycle of poverty. The above case highlights some of the problems faced by developing countries in tackling the neglected tropical diseases, with the recent conflict in South Sudan only serving to amplify this problem. The approach to addressing the problem of the neglected tropical diseases must be multi-focused, with both the development of new treatments and ensuring effective prevention schemes. Guinea worm, an all but eradicated disease, provides an excellent example of an effective public health scheme using simple control techniques [8, 9]. Current control schemes, including anti-helminthic treatment of dogs, health education and quarantine have in some states been shown to be an effective means of control of CE [10]. However several national programmes, mostly in non-island nations, have been unsuccessful in reducing the burden of CE [11]. Such prevention schemes are costly and logistically difficult in countries with already struggling health systems. Furthermore with its current political situation, implementing these strategies in South Sudan becomes increasingly more difficult. The possibility of using canine and bovine vaccines to help curb transmission of E. Granulosa has yielded promising initial results, and could hold the key to potential eradication of the disease in the future [12, 13].

## Conclusion

The impact of CE is felt throughout the world, with considerable economic and health implications for individuals and society as a whole. In South Sudan and other developing nations, lack of imaging tools, surgeons trained in cyst removal and access to appropriate medications have resulted in mismanagement and under-diagnosis of established hydatid disease. For this reason, hydatid disease should be included in the differential diagnosis for all patients with abdominal pain from this region. Further research and government lead prevention initiatives are required to help reduce the burden of the disease.
